# Machine learning helps improve diagnostic ability of subclinical keratoconus using Scheimpflug and OCT imaging modalities

**DOI:** 10.1186/s40662-020-00213-3

**Published:** 2020-09-10

**Authors:** Ce Shi, Mengyi Wang, Tiantian Zhu, Ying Zhang, Yufeng Ye, Jun Jiang, Sisi Chen, Fan Lu, Meixiao Shen

**Affiliations:** 1 School of Ophthalmology and Optometry, Wenzhou Medical University, 270 Xueyuan Road, Wenzhou, Zhejiang, 325027 China; 2 College of Computer Science and Technology, Zhejiang University of Technology, Hangzhou, Zhejiang 12624 China

**Keywords:** Subclinical keratoconus, Machine learning, Combined-devices, Ultra-high resolution optical coherence tomography, Scheimpflug camera

## Abstract

**Purpose:**

To develop an automated classification system using a machine learning classifier to distinguish clinically unaffected eyes in patients with keratoconus from a normal control population based on a combination of Scheimpflug camera images and ultra-high-resolution optical coherence tomography (UHR-OCT) imaging data.

**Methods:**

A total of 121 eyes from 121 participants were classified by 2 cornea experts into 3 groups: normal (50 eyes), with keratoconus (38 eyes) or with subclinical keratoconus (33 eyes). All eyes were imaged with a Scheimpflug camera and UHR-OCT. Corneal morphological features were extracted from the imaging data. A neural network was used to train a model based on these features to distinguish the eyes with subclinical keratoconus from normal eyes. Fisher’s score was used to rank the differentiable power of each feature. The receiver operating characteristic (ROC) curves were calculated to obtain the area under the ROC curves (AUCs).

**Results:**

The developed classification model used to combine all features from the Scheimpflug camera and UHR-OCT dramatically improved the differentiable power to discriminate between normal eyes and eyes with subclinical keratoconus (AUC = 0.93). The variation in the thickness profile within each individual in the corneal epithelium extracted from UHR-OCT imaging ranked the highest in differentiating eyes with subclinical keratoconus from normal eyes.

**Conclusion:**

The automated classification system using machine learning based on the combination of Scheimpflug camera data and UHR-OCT imaging data showed excellent performance in discriminating eyes with subclinical keratoconus from normal eyes. The epithelial features extracted from the OCT images were the most valuable in the discrimination process. This classification system has the potential to improve the differentiable power of subclinical keratoconus and the efficiency of keratoconus screening.

## Background

The accurate identification of keratoconus (KC) at its earliest stage is the primary concern in corneal refractive surgery preoperative screening for several reasons. Corneas with undetected KC are known to be highly associated with iatrogenic keratectasia, which is the most severe and irreversible complication after laser in situ keratomileusis (LASIK) [1, 2]. In addition, with the availability of therapies such as corneal cross-linking, early detection can also contribute to delaying or stopping the progression of KC [3]. However, KC identification can be challenging clinically in its early stages because visual acuity remains good and there is no specific corneal finding.

Keratoconus can be well defined and easily detected through slit-lamp biomicroscopy and corneal Placido reflection-based topography [4]. However, the definition of subclinical KC itself is ambiguous [5]. The information acquired from traditional imaging methods is limited, and using these methods, the diagnostic capacity is insufficient in identifying subclinical KC. Recently, new ophthalmic imaging modalities have been applied in the screening of KC at its earliest stage [6, 7]. Among these modalities, Scheimpflug-based camera imaging and spectral domain optical coherence tomography (SD-OCT) have been the most widely studied methods. Both approaches have provided unique imaging advantages in recognizing early changes in the cornea (e.g., depth information, corneal microstructures, etc.) and have been proven to provide diagnostic value in detecting subclinical KC [5]. Hwang et al. reported a direct statistical approach using a mixed topography variable from a Scheimpflug-based camera and SD-OCT that reached high discrimination [8]. However, in clinical settings, combined machine-derived parameters from these instruments are often too complicated for clinicians to interpret.

This dilemma can possibly be addressed by the advent of artificial intelligence (i.e., machine learning). The use of artificial intelligence in corneal topography has a history of over a decade [9]. However, the early applications of machine learning in corneal topography were restricted to a single machine or several metrics derived from the same image; hence, the diagnostic ability of these models to detect subclinical KC relied on a large sample size [10–12]. In light of the good performance of combined tomography instruments in previous studies, an automated screening approach using machine learning may dramatically help clinicians classify subclinical KC.

In this study, we present an automated classification system using the combination of Scheimpflug camera and UHR-OCT imaging parameters based on a machine learning classifier to distinguish a population with subclinical keratoconus from a normal control population. We report that the machine learning-derived classifier can provide valuable identification of subclinical KC. Moreover, multiple machines that combine features demonstrate better performance than a single machine that derives features.

## Method

The study was approved by the Ethics Committee of the Eye Hospital of Wenzhou Medical University (ID: Y-2015003) and adhered to the tenets of the Declaration of Helsinki. Written informed consent was obtained from each subject.

### Study population

A total of 121 eyes of 121 subjects were examined between September 2015 and July 2018. The demographic characteristics of all enrolled subjects are shown in Table 1. All subjects were imaged with the Pentacam HR system (Oculus, Gmbh, Wetzlar, Germany) and a UHR-OCT prototype system. Patients with KC (Group 1) and subclinical KC (Group 2) were recruited from the Affiliated Eye Hospital of Wenzhou Medical University. Normal subjects (Group 3) were recruited from the hospital’s working staff and students. A comprehensive ocular exam was performed by experienced doctors (YY and JJ) and included a review of family and medical history, corrected-distance visual acuity, slit-lamp biomicroscope examination, fundus examination and corneal topography (Medmont, Inc., Nunawading Melbourne, Australia). The subjects were assigned to one of three groups.

**Table 1:** Demographic characteristics of normal, subclinical keratoconus, and keratoconus groups

	Normal (*n* = 50)	Sub KC (*n* = 33)	KC (*n* = 38)
SE (D)	−3.9 ± 2.3	−3.7 ± 4.0	−6.7 ± 4.5*
BCVA (decimal VA)	1.1 ± 0.1	1.0 ± 0.2*	0.5 ± 0.3*
Max-K (D)	43.0 ± 1.3	43.1 ± 1.9	46.3 ± 3.3*
Min-K (D)	44.3 ± 1.6	44.7 ± 1.3	51.2 ± 4.7*
Avg-K (D)	43.7 ± 1.4	43.9 ± 2.0	48.8 ± 3.6*
Ast-K (D)	1.3 ± 0.7	1.6 ± 0.9	5.3 ± 3.1*

*Normal*= normal group; *Sub-KC*= subclinical keratoconus group; *KC*= keratoconus group; *n*= number of eyes; *SE*= spherical equivalent; *BCVA*= best corrected visual acuity; *Max-K*= maximum keratometry; *Min-K*= minimum keratometry; *Avg-K*= average keratometry; *Ast-K*= astigmatic keratometry; *VA*= visual acuity; *D*= diopter; *P < 0.05 compared to the normal group

Group 1. One eye of each patient with KC was included in this study. The KC patients were diagnosed by the following clinical findings: (1) the presence of at least one of the following slit-lamp signs: Vogt’s striae, stromal thinning, Fleischer’s ring > 2 mm arc; (2) a central average keratometry above 47.0 D; (3) asymmetric topographical features with inferior-superior (I-S) values above or equal to 2.0 D of the vertical power gradient across the 6-mm region; (4) no history of contact lens use, ocular surgery or extensive scarring.

Group 2. Subclinical KC eyes were identified from the other eyes of unilateral KC patients, and patients meeting all criteria mentioned below were recruited: (1) no clinical signs of KC during slit-lamp biomicroscope examination, retinoscopy and ophthalmoscopy; (2) a diagnosis of KC in the contralateral eye; (3) a central average keratometry less than 45.0 D; (4) corneal topographical features with I-S values less than 1.4 D of the vertical power gradient across the 6-mm region; (5) myopia less than – 6.0 D with astigmatism less than − 2.0 D; (6) no history of contact lens wear or ocular surgery.

Group 3. Normal eyes were included if they met the following criteria: (1) no clinical signs or suggested suspected subclinical KC or KC patterns from corneal topography images; (2) a central average keratometry less than 45.0 D; (3) I-S values less than 1.4 D of the vertical power gradient across the 6-mm region; (4) myopia less than − 6.0 D and astigmatism less than − 2.0 D; (5) no history of contact lens wear, ocular surgery or trauma.

### Scheimpflug-based imaging acquisition procedure

**Fig. 1:**
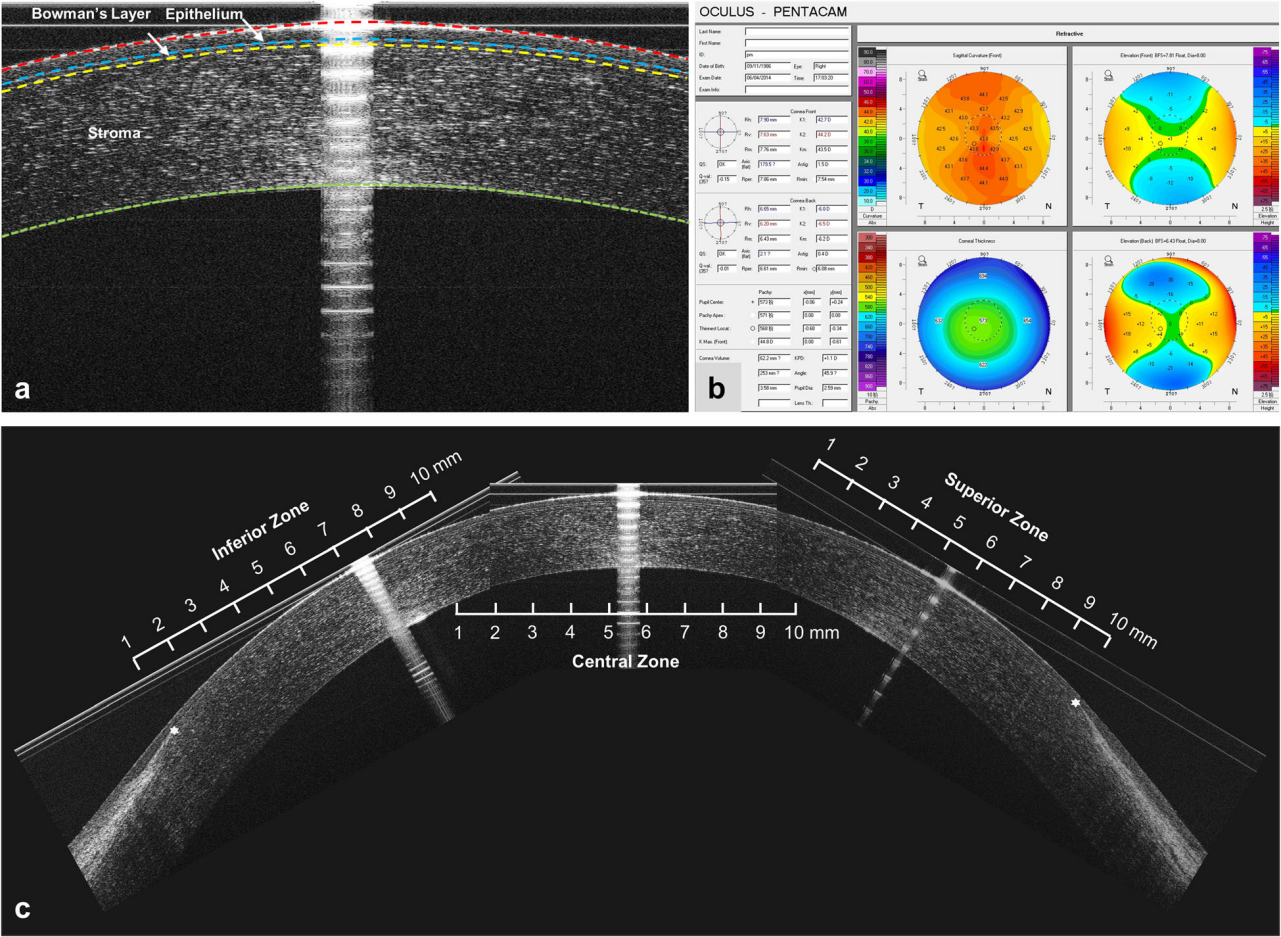
Representative UHR-OCT images and Pentacam HR system report. **a** Representative UHR-OCT image of a normal cornea. The cornea was automatically segmented into three layers (epithelium, Bowman’s layer and stroma). **b** Representative Pentacam HR system report of normal eye. Parameters were extracted from the report. **c** Reconstruction of the entire corneal profile. Each region was divided into 10 equal zones to perform data analysis, and the superior and inferior zones ended at the edges of Bowman’s layer (*)

A Pentacam HR system (Oculus, Gmbh, Wetzlar, Germany) was used to perform the corneal tomographic examinations (Fig. 1b). All procedures were performed by an experienced operator, and all participants were asked to blink once before image acquisition. Only when “Examination Quality Specification” showed “OK” were the corneal curvature, elevation and pachymetry results accepted. A total of three repeated measurements were performed on each subject. The built-in Pentacam HR software (version 6.02r23) was used to export the machine-based metrics, including metrics from the elevation and curvature values from the anterior and posterior interfaces as well as corneal pachymetry mapping. The average value obtained from three measurements on the same subject was recorded.

### UHR-OCT imaging acquisition procedure

A prototype UHR-OCT system was used to acquire corneal images, which have been described previously (Fig. 1a and c) [7, 13, 14]. In brief, the UHR-OCT system used a three-module superluminescent diode (SLD) light source (Broadlighter, T840-HP, Superlumdiodes Ltd., Moscow, Russia) with a center wavelength of 840 nm and a full width at half maximum bandwidth of 100 nm, which had approximately 3 μm of axial resolution in corneal tissue with a scan speed of 24 k A-lines per second. The image width was 8.66 mm. The imaging procedure was performed by an experienced operator working from 9 AM to 5 PM. Central images were acquired by guiding each participant to stare at the internal visual target positioned in front of the eye for alignment. Superior and inferior images were acquired by guiding the subject to stare at the external fixation target positioned 15 cm from the subjects, with 30° upward and 30° downward angles. Custom developed software based on MATLAB 2018a (MathWorks, Inc., Natick, MA, USA) was used to perform image analysis, in which the thickness profiles of the epithelium, Bowman’s layer and stroma were automatically extracted for further analysis [7].

### Analyzed parameters and description

Both eyes of all participants were imaged by the Pentacam HR system and UHR-OCT, and only one eye of the normal control subjects and KC patients was randomly selected for analysis. A total of 49 parameters were extracted. All parameters were independent variables. Briefly, the analyzed parameters were described as follows:

#### Pentacam HR system curvature-based parameters:

- Flat keratometry (K1): K1 represented the flat corneal curvature in the central 3.0 mm zone.
- Steep keratometry (K2): K2 represented the steep corneal curvature in the central 3.0 mm zone.
- Average keratometry (Km): Km represents the average values of K1 and K2.
- Steepest keratometry (Kmax): Kmax represents the steepest corneal curvature in the cornea.

#### Pentacam HR system elevation-based parameters:

Elevation maps were generated with the 8 mm best-fit sphere (BFS) float mode. Elevation values were manually detected in the central 5 mm area in both the front and back corneal surfaces. Four elevation values were recorded with the average values of 3 repeated measurements:

1. Max elevation (Emax (front) and Emax (back)): the maximum elevation of the front or back surface.
2. Central elevation (Ecenter (front) and Ecenter (back)): the elevation at the cornea apex of the front or back surface.

#### Pentacam HR system pachymetry-based parameters:

Two parameters calculated over a diameter of 8.0 mm were recorded:

1. Thinnest point: the thickness value at thinnest point of the cornea
2. Corneal volume: the volume of the cornea with a diameter of 8 mm, centered on the anterior corneal apex.

#### Pentacam HR system integrated parameters:

Seven parameters were exported from Pentacam HR built-in software.

(1) ISV: index of surface variance; (2) IHA: index of height asymmetry; (3) IVA: index of vertical asymmetry; (4) IHD: index of height decentration; (5) KI: keratoconus index; (6): Rmin: smallest radius; (7): CKI: central keratoconus index.

#### UHR-OCT system epithelium, Bowman’s layer and stroma pachymetry-based parameters:

- Average thickness (EMean, BMean, SMean): EMean, BMean and SMean represent the average thickness of the epithelium, Bowman’s layer and stroma, respectively, in different locations (total, superior and inferior).
- Minimum thickness (Emin, Bmin, Smin): Emin, Bmin and Smin represent the thinnest thickness of the inferior thickness map of the epithelium, Bowman’s layer and stroma.
- Maximum thickness (Emax, Bmax, Smax): Emax, Bmax and Smax represent the thickest thickness of the superior thickness map of the epithelium, Bowman’s layer and stroma.
- Ectasia index (EEI, BEI and SEI): EEI, BEI and SEI represent localized thinning in the vertical meridian of the epithelium, Bowman’s layer and stroma. The index was defined as the minimum thickness in the inferior half divided by the average thickness in the superior half multiplied by 100.
- Maximum ectasia index (EEI-MAX, BEI-MAX and SEI-MAX): EEI-MAX, BEI-MAX and SEI-MAX represent the maximum localized thinning in the vertical meridian of the epithelium, Bowman’s layer and stroma. The index was defined as the minimum thickness in the inferior half divided by the maximum thickness in the superior half multiplied by 100.
- Profile variation (EPV, BPV and SPV): EPV, BPV and SPV represent the variation of thickness profile within each individual of the epithelium, Bowman’s layer and stroma. It was defined as the root mean square between the zone thickness and the profile average within one subject.
- Profile deviation (EPSD, BPSD and SPSD): EPSD, BPSD and SPSD represent the standard deviation of the thickness profile between individual and normal patterns of the epithelium, Bowman’s layer and stroma. It was defined as the root mean square of the zonal thickness of the individual profiles and zonal thicknesses of the pattern average.

#### Other parameters

Gender.

### Automated machine learning classifier

**Fig. 2:**
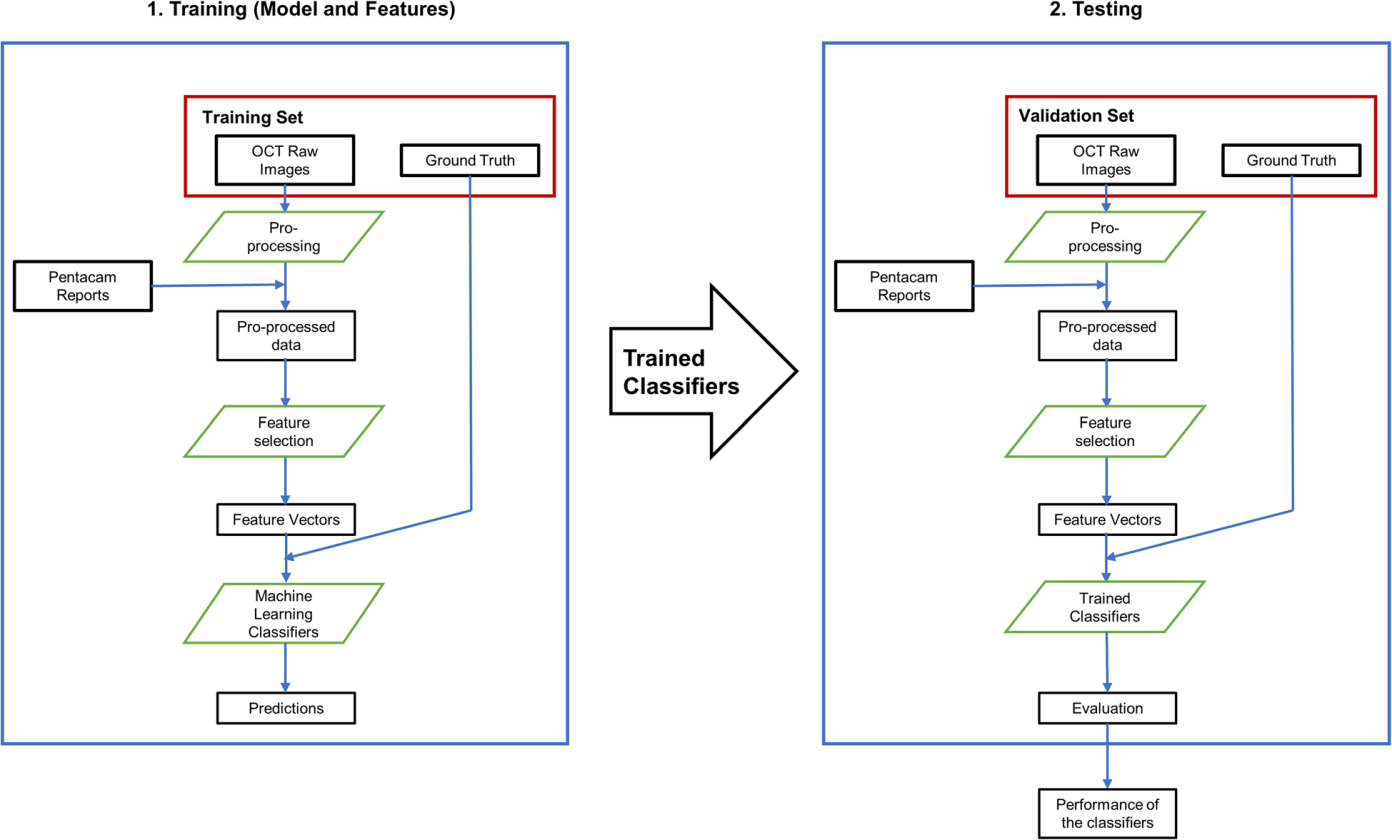
Workflow of training and validate machine learning classifier

In our study, all machine learning classifiers were built in an open-source Python package with Python 3.5 (Python Software Foundation, https://www.python.org). The classifier was used to discriminate normal, subclinical KC, and KC corneas in an objective and quantitative way. The workflow is detailed in Fig. 2. In short, 70% of the cases were randomly selected, and 30% of the cases were divided into training and validation sets. All data were normalized before training. When the logistic regression and neural network classifier reached the highest sensitivity and specificity, the resulting model was selected as the automated machine learning classifier. The whole procedure was repeated for 100 times; the receiver operating characteristic (ROC) curves were calculated each time to obtain the area under the ROC curves (AUCs), and sensitivity and specificity were calculated separately in the validation sets.

### Fisher’s scoring system

We manually chose 49 parameters from the three different datasets (normal, subclinical KC, and KC eyes). A feature selection procedure was used to gain a better understanding of all the features and reduce the overfitting of the classifier model caused by some absolute features [15]. We used Fisher’s score to evaluate the discriminative power of each feature. We listed the correlation between the Fisher score and the feature’s impact on the classification accuracy for all features. If the corresponding feature has no discriminative power among different eyes, then the Fisher score will be close to zero, and the classification average accuracy of the feature will be low. In contrast, selected features are considered good if their Fisher scores are much larger than zero. Classification with these features can achieve high accuracy. Consequently, we selected the top 5 features according to the Fisher score of each classifier for a detailed presentation (Table 2).

**Table 2:** List of parameters measured by the Pentacam HR System, UHR-OCT and submitted to the neural network classifier for discriminating sub KC and KC corneas from normal healthy corneas

Pentacam HR System Parameters				UHR-OCT Parameters		
Curvature-Derived Parameters	Elevation-Derived Parameters	Pachymetry-Derived Parameters	Integrated Parameters	OCT-Derived Parameters		
**Anterior surface:**	**Anterior surface:**	Thinnest point	ISV	**Epithelium**	**Bowman’s Layer**	**Stroma**
K1 (Front)	Emax (Front)	Corneal Volume	IHA	EPSD	BPSD	SPSD
K2 (Front)	Ecenter (Front)		IVA	EPV	BPV	SPV
Km (Front)			IHD	EEI (I/S)	BEI(I/S)	SEI(I/S)
Kmax (Front)			KI	EEI-MAX (I/S)	BEI-MAX (I/S)	SEI-MAX (I/S)
**Posterior surface:**	**Posterior surface:**		Rmin	EMean (total)	BMean (total)	SMean (total)
K1 (Back)	Emax (Back)		CKI	Emean (I)	Bmean (I)	Smean (I)
K2 (Back)	Ecenter (Back)			Emean (S)	Bmean (S)	Smean (S)
Km (Back)				Emin (I)	Bmin (I)	Smin (I)
Kmax (Back)				Emax (S)	Bmax (S)	Smax (S)

*UHR-OCT*= ultra-high resolution optical coherence tomography; *Sub KC*= subclinical keratoconus; *KC*= keratoconus; *K1*= flattest keratometric reading; *K2*= steepest keratometric reading; *Km*= mean keratometric reading; *Kmax*= maximum keratometric reading; *Emax*= maximum elevation reading; *Emin*= minimum elevation reading; *Ecenter*= corneal central elevation reading; *ISV*= index of surface variance; *IHA*= index of height asymmetry; *IVA*= index of vertical asymmetry; *IHD*= index of height decentration; *KI*= keratoconus index; *Rmin*= smallest radius; *CKI*= central keratoconus index; EPSD, BPSD, SPSD: standard deviation of thickness profile between individual and normal pattern of epithelium, Bowman’s layer and stroma; EPV, BPV, SPV: profile variation of epithelium, Bowman’s layer or stroma thickness profile within each individual; EEI (I/S), BEI (I/S), SEI (I/S): ectasia index of epithelium, Bowman’s layer or stroma; EEI-MAX (I/S), BEI-MAX (I/S), SEI-MAX (I/S): Maximum ectasia index of epithelium layer, Bowman’s layer or stroma; EMean (total); BMean (total); SMean (total): mean thickness of epithelium, Bowman’s layer or stroma; EMean (I), Bmean (I), Smean (I): mean inferior thickness of epithelium; Bowman’s layer or stroma; EMean (S), Bmean (S), Smean (S): mean superior thickness of epithelium; Bowman’s layer or stroma; Emin (I), Bmin (I), Smin (I): the thinnest thickness of the inferior epithelium; Bowman’s layer or stroma thickness map; Emax(S), Bmax(S), Smax (S): the thickest thickness of the superior epithelium; Bowman’s layer or stroma thickness map

### Statistical method

SPSS software (version 22.0; SPSS, Inc., Chicago, IL, USA) was used for all statistical procedures. Data for continuous features are presented as mean ± standard deviation. Student’s t-tests were used to compare corneal features in normal subjects, subclinical KC subjects and KC subjects. *P*-values of less than 0.05 were considered statistically significant.

## Results

### Logistic regression classifier and neural network classifier discriminating power and each variable discriminating power

#### Normal vs subclinical KC

**Fig. 3:**
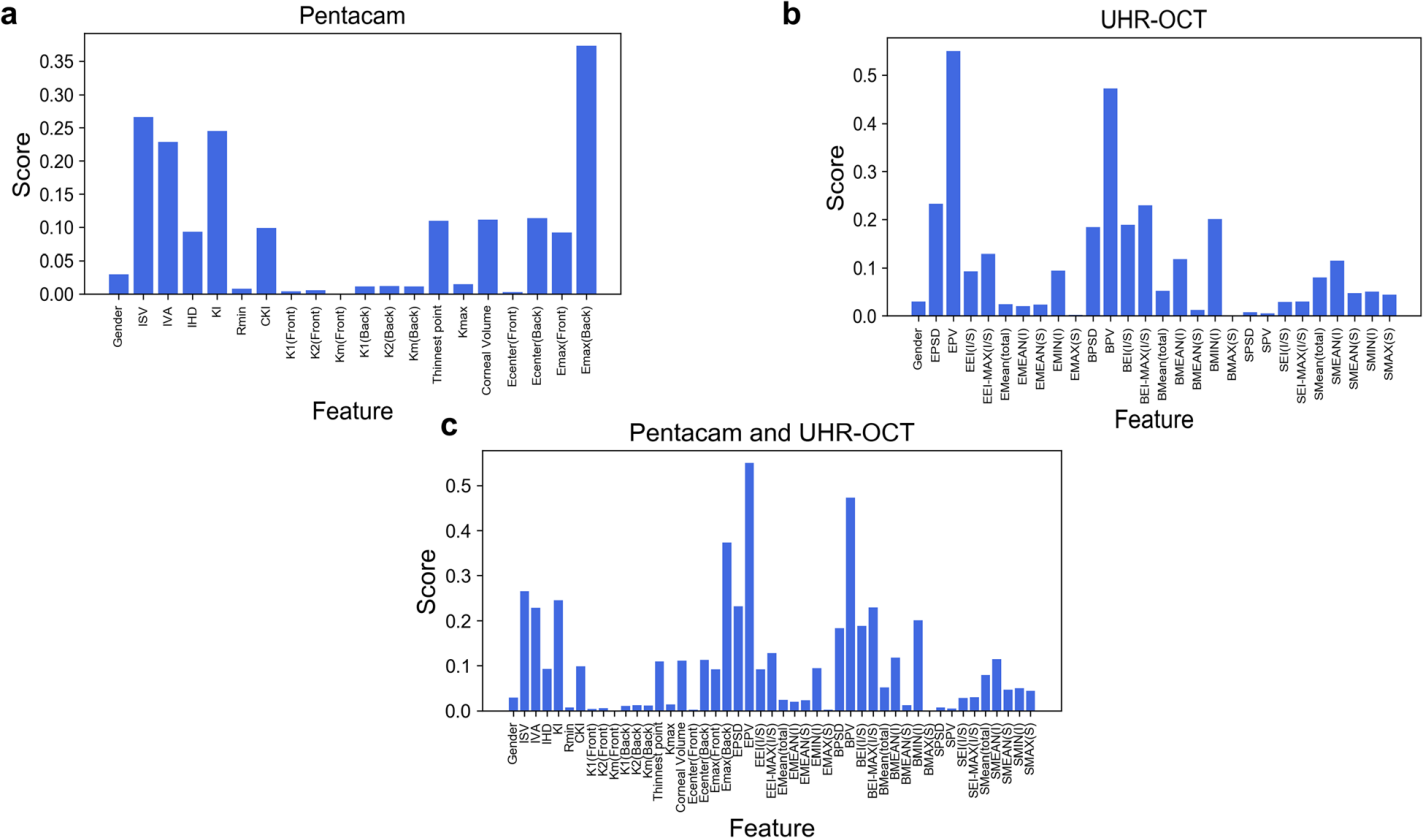
Fisher’s score of each variable of different classifiers to discriminate subclinical KC eyes from normal eyes. For subclinical KC eyes, using the Pentacam HR system alone, the features contributing to discrimination most were the maximum elevation values in the 5 mm area (**a**). Using UHR-OCT alone or combining it with the Pentacam HR system, the variable that contributed to discrimination most by ranking was EPV (**b**, **c**). KC: keratoconus. UHR-OCT: Ultra-high-resolution optical coherence tomography; EPV: epithelium profile variation

Using the Pentacam HR system alone or UHR-OCT alone, the logistic regression classifier showed good discriminating power, reaching an AUC = 0.74 (Pentacam HR system) and an AUC = 0.90 (UHR-OCT); the neural network classifier reached an AUC = 0.68 (Pentacam HR system) and an AUC = 0.88 (UHR-OCT). After combining features from the Pentacam HR system and UHR-OCT, the classifier reached an AUC = 0.90 for the logistic regression classifier. For the neural network classifier, the AUC was 0.93 (Table 3). Using the Pentacam HR system alone, by ranking Fisher’s score, the variable that contributed to discrimination most was Emax (Back) (Fig. 3a). Using UHR-OCT alone or combining the UHR-OCT with the Pentacam HR system, the feature that contributed to discrimination most by ranking was EPV (Fig. 3b and c). We listed the detailed information of the top 5 features that contributed the most to the classifier in Table 4.

**Table 3:** Performance of the discriminating rules generated using logistic regression and neural network classifiers for differentiating sub KC and KC corneas from normal corneas

	Normal vs. Sub KC			Normal vs. KC		
	Sensitivity	1-Specificity	AUC	Sensitivity	1-Specificity	AUC
**Logistic Regression**						
Pentacam HR system	83.8%	88.7%	0.74	100%	100%	1.00
UHR-OCT	95.3%	94.5%	0.90	98.0%	100%	0.98
Pentacam HR system & UHR-OCT	95.1%	94.8%	0.90	100%	99.4%	0.99
**Neural Network**						
Pentacam HR system	82.1%	82.6%	0.68	100%	100%	1.00
UHR-OCT	94.8%	93.4%	0.88	99.5%	99%	0.98
Pentacam HR system & UHR-OCT	98.5%	94.7%	0.93	100%	100%	1.00

*UHR-OCT*= ultra-high resolution optical coherence tomography; *Sub KC*= subclinical keratoconus group; *KC*= keratoconus group

**Table 4:** Demographics of top 5 variables listed in the Fisher’s scoring system using different variables from Pentacam HR model and UHR-OCT model to discriminate sub-clinical KC group from normal group

	Mean ± SD			Intragroup Comparison	
	Normal	Sub KC	KC	Normal vs. Sub KC	Normal vs. KC
Features				P	
**Pentacam & UHR-OCT Model**					
EPV (μm) ^b^	2.8 ± 0.7	4.1 ± 1.0	6.8 ± 2.5	< 0.001	< 0.001
BPV (μm) ^b^	1.3 ± 0.3	1.7 ± 0.5	2.6 ± 1.0	< 0.001	< 0.001
EMax (back) (mm) ^a^	4.8 ± 2.5	11.1 ± 7.4	28.5 ± 10.3	< 0.001	< 0.001
ISV ^a^	17.2 ± 5.9	24.5 ± 8.3	91.0 ± 37.2	< 0.001	< 0.001
KI ^a^	1.02 ± 0.03	1.05 ± 0.03	1.21 ± 0.12	< 0.001	< 0.001
**Pentacam Model**					
IVA	0.1 ± 0.1	0.2 ± 0.1	0.8 ± 0.4	< 0.001	< 0.001
Ecenter (back) (mm)	−0.6 ± 2.2	1.9 ± 5.0	14.7 ± 10.6	0.012	< 0.001
**UHR-OCT Model**					
EPSD (μm)	3.3 ± 1.1	4.4 ± 1.2	7.9 ± 2.4	< 0.001	< 0.001
BEI-MAX (I/S) (μm)	78.6 ± 5.4	63.6 ± 23.4	50.0 ± 14.4	0.001	< 0.001
BMIN (I) (μm)	15.2 ± 1.5	12.4 ± 4.5	9.4 ± 2.6	0.001	< 0.001

^a^ Included in the UHR-OCT model. ^b^ Included in the Pentacam model. *Sub KC*= subclinical keratoconus group; *KC*= keratoconus group; *UHR-OCT*= ultra-high resolution optical coherence tomography; *EPV*= profile variation of epithelium; *BPV*= profile variation of Bowman’s layer; Emax (back): max elevation of 5 mm best-fit sphere of back corneal surface. *ISV*= index of surface variance; *KI*= keratoconus index; *IVA*= index of vertical asymmetry; Ecenter (back): central elevation of 5 mm best-fit sphere of back corneal surface; EPSD = epithelium profile standard deviation; BEI-MAX: maximum ectasia index of Bowman’s layer; Bmin (I): the thinnest thickness of the inferior Bowman’s layer thickness map

#### Normal vs KC

**Fig. 4:**
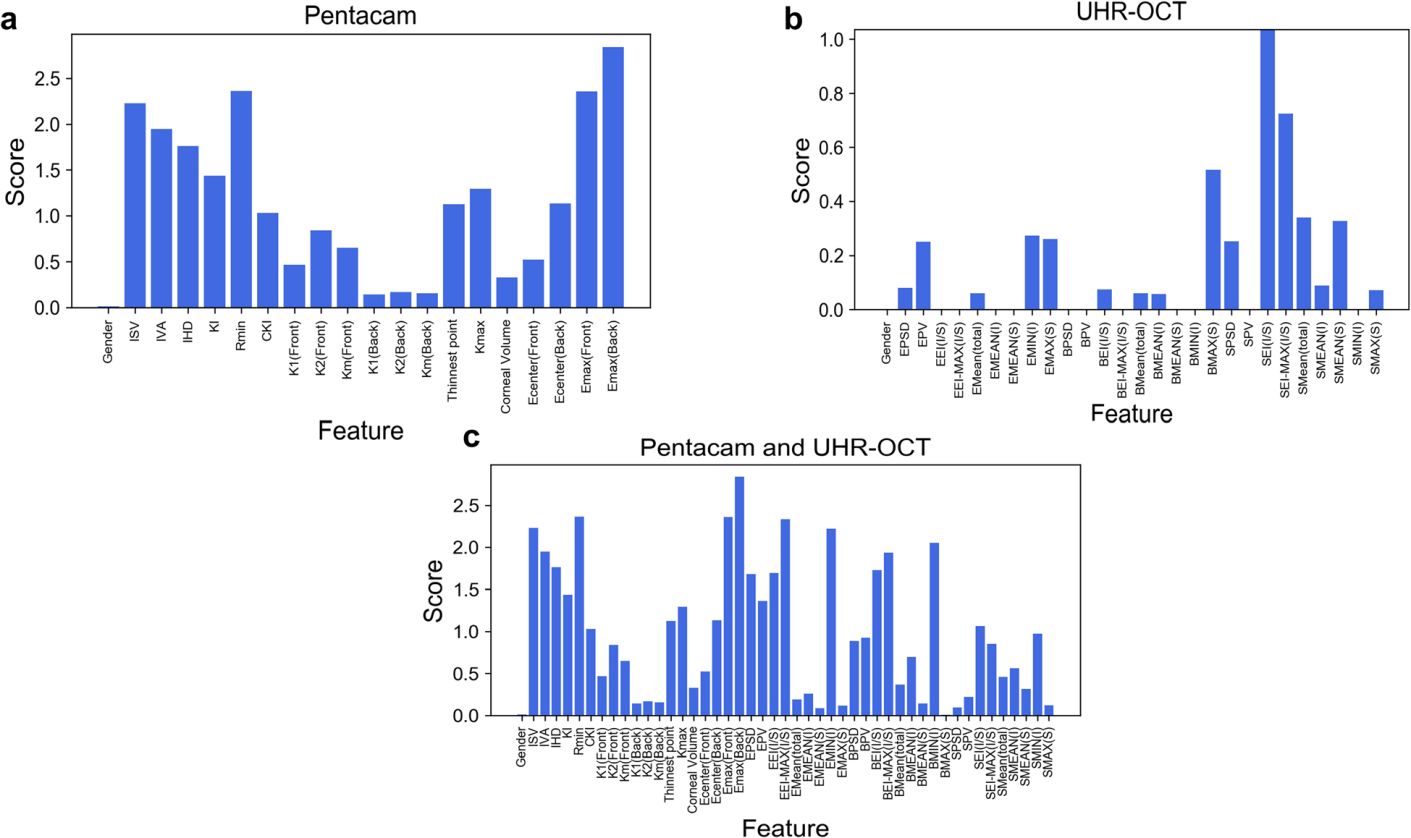
Fisher’s score of each variable of different classifiers to discriminate KC eyes from normal eyes. For KC eyes, using the Pentacam HR system alone and combining it with UHR-OCT, the feature that contributed to discrimination most by ranking was the maximum elevation value in the 5 mm area (**a**, **c**). Using UHR-OCT alone, the feature that contributed to discrimination most by ranking was SEI (I/S) (**b**). KC: keratoconus. UHR-OCT: Ultra-high-resolution optical coherence tomography; SEI (I/S): Localized thinning in the vertical meridian in the stroma

Both classifiers showed great discriminating power using the Pentacam HR system alone (Both AUCs = 1.0), UHR-OCT alone (Bothe AUCs = 0.98) or combined Pentacam HR system and UHR-OCT, and the AUC of the logistic regression classifier is 0.99 and for the neural network classifier is 1.00 (Table 3). Whether using the Pentacam HR system alone or combined with the UHR-OCT, by ranking Fisher’s score, the variable that contributed the most to discrimination was Emax (Back) (Fig. 4a and c). Using UHR-OCT alone, the variable that contributed the most to discrimination by ranking was SEI (I/S) (Fig. 4b).

## Discussion

Our study demonstrated that machine learning-derived classifiers provide good differential power to differentiate subclinical KC eyes from normal eyes. Multiple instrument-combined variables achieved better performance than single instrument-derived variables. To differentiate subclinical KC eyes from normal eyes, UHR-OCT variables contributed more than the Scheimpflug-based camera variables. To the best of our knowledge, this is the first study using combined SD-OCT and Scheimpflug-based camera variables through a machine learning classifier to differentiate subclinical KC eyes from normal eyes and compare the differentiation power of each variable.

Typical KC signs, such as Fleischer’s Ring and stromal thinning, can be easily observed by a slit-lamp biomicroscope [16]. The abnormally high K value or I-S value detected by Placido ring-based corneal tomography can also indicate abnormal KC corneas [17]. These indices are widely recognized among clinicians. However, the nature of these instruments limits their sensitivity to detect subtle changes in the cornea. In contrast, Scheimpflug-based cameras can acquire multiple corneal morphology information, including both anterior and posterior curvature and elevation and regional corneal thickness [18]. In addition, UHR-OCT can achieve high resolution in corneal tissues, providing information on corneal microstructure in depth [19]. The advantages of requiring detailed information were reflected on our results; combined Scheimpflug-based camera and UHR-OCT variables reached excellent precision (0.98) to differentiate KC eyes from normal eyes, even with a single machine. However, for most clinicians, the real challenge is to diagnose subclinical KC early.

Unlike KC, the identification of subclinical KC is often challenging in routine clinical practice, as it is usually asymptomatic and is considered the most significant risk factor for the development of iatrogenic ectasia after LASIK [2]. Our previous studies reported that the use of epithelium and Bowman’s layer features extracted from UHR–OCT [7] or the parameters from the Pentacam HR system alone [20] can reach good differential power for subclinical KC detection. However, because of the difficulty in correcting the optical distortion of OCT images, the OCT system lacks topography indices. Additionally, due to the resolution limitations of the Pentacam HR system, the Pentacam camera cannot accurately detect corneal sublayers. Our current study used a similar approach and reached good differential power for subclinical KC detection. Hence, combining the features of the Pentacam HR system and UHR-OCT will help us fully understand the subtle structural changes in subclinical KC eyes and better differentiate them from normal eyes.

Attempts have been made to utilize combined instruments and demonstrate good differentiation power compared to single instruments. Amobrosio et al. studied combined Scheimpflug-based corneal tomography and biomechanics and found enhanced corneal ectasia detection [21]. This study indicated the potential application of multiple instrument-derived variables in diagnosing subclinical KC. Recently, Hwang et al. used multivariable analysis, achieving great diagnostic power of subclinical KC corneas using combined SD-OCT and Scheimpflug-based camera variables (AUC = 1.0) [8]. However, the inclusion criteria of subclinical KC subjects for that study were controversial, and the processing of these indices was time consuming and not user friendly for clinical application [22, 23]. We used a logistic regression classifier to differentiate subclinical KC in this study; however, the precision accuracy was not improved compared to a single machine-derived logistic regression model. When the feature number is large, the logistic regression classifier is prone to underfitting, and the prediction accuracy will consequently be limited in this scenario. A machine-learning-based model can avoid this issue. Hence, machine learning (ML) models, such as neural networks, are more appropriate when multiple instruments are used. Our results showed that the AUCs of the neural network classifier was higher than that of the logistic regression classifier.

Neural networks, as machine learning classifiers, have multiple advantages, such as self-learning and being free from data loss, and similar approaches have been applied to KC diagnosis for a few years. Smolek et al. introduced a neural network approach using corneal topographic variables to detect KC and KC suspected eyes. The neural network approach demonstrated superior accuracy to the manual screening approach [9]. Other machine learning classifiers have also been applied to KC diagnosis. Arbelaez et al. used both anterior and posterior variables from Scheimpflug-based cameras and corneal topography, and the SVM classifier had a differential power of 0.92 for subclinical KC eyes [11]. Smadja et al. used variables from Scheimpflug-based cameras and automated decision-tree classification to detect subclinical KC eyes and achieved excellent sensitivity (93.6%) and specificity (97.2%). We summarized different machine learning classifier applications in KC diagnosis in Table 5 and compared our results with those of other studies. We found that in our study, the automated classifier based on the Scheimpflug-based camera and UHR-OCT achieved similar differential power compared to other studies with a smaller sample size. The results indicated that sublayer information of the cornea derived from the UHR-OCT and multiple features derived from the Pentacam HR system were useful for differentiating subclinical KC eyes from normal eyes. These successful applications of machine learning-based classifiers and the high differential power indicated that machine learning-based automated classification systems are a powerful tool for screening subclinical KC eyes.

**Table 5:** Summary of studies using machine learning classifier for different KC or subclinical KC eyes from normal eyes

Authors	Year	Instruments	ML classifier	Subjects	Results
Current Study	2019	UHR-OCT, Scheimpflug camera	Neural network	38 eyes with KC, 33 eyes with subclinical KC, 50 normal eyes	93% precision for subclinical KC eyes, 99% precision for KC eyes
Smolek et al. [9]	1997	Corneal topography	Neural network	6 KC suspect eyes, 33 eyes with KC	100% accuracy, sensitivity and specificity for all KC suspect and KC eyes
Accardo et al. [24]	2002	Corneal topography	Neural network	120 eyes with early KC eyes, 120 normal eyes	94.1% sensitivity, 97.6% specificity for early KC eyes
Arbelaez et al. [11]	2012	Scheimpflug camera and Placido corneal topography	SVM	877 eyes with KC, 426 eyes with subclinical KC, 1259 healthy control eyes	98.2% accuracy (95.0% sensitivity and 99.3% specificity) for KC eyes and 97.3% accuracy (92.0% sensitivity and 97.7% specificity) for subclinical KC eyes
Smadja et al. [10]	2013	Scheimpflug camera	Decision tree	148 eyes with KC, 177 eyes with forme fruste KC, 372 healthy control eyes	100% sensitivity and 99.5% specificity for KC eyes, 93.6% sensitivity and 97.2% specificity for forme fruste KC eyes
Kovacs et al. [25]	2016	Scheimpflug camera	Neural network	60 eyes with KC, 15 eyes with preclinical KC, 60 healthy control eyes	0.99 AUC, 100% sensitivity and 98% specificity for KC eyes, 0.96 AUC, 92% sensitivity and 85% specificity for preclinical KC eyes
Saad et al. [26]	2016	Placido based corneal topography and corneal wavefront measurements	Neural network	62 eyes with forme fruste KC, 114 normal eyes	0.97 AUC, 63% sensitivity and 82% for forme fruste KC, 100% sensitivity and 82% specificity for KC eyes
Hidalgo et al. [27]	2016	Scheimpflug camera	SVM	454 eyes with KC, 67 eyes with forme fruste KC, 194 normal eyes	98.9% accuracy, 99.1% sensitivity and 98.5% specificity for KC eyes, 93.1% accuracy, 79.1% sensitivity and 97.7% specificity for forme fruste KC eyes
Ambrosio et al. [21]	2017	Scheimpflug camera and biomechanical camera	SVM, random forest	111 eyes with KC, 227 normal eyes	1.0 AUC for KC eyes
Lopes et al. [12]	2018	Scheimpflug camera	Random forest	71 eyes with ectasia susceptibility, 182 eyes with KC, 2980 normal eyes	85.2% sensitivity and 0.966 specificity, 0.968 AUC for suspected KC eyes.
Issarti et al. [28]	2019	Scheimpflug camera	Neural network	77 eyes with suspect KC, 312 normal eyes	96.56% accuracy, 97.78% sensitivity and 95.56% specificity for suspect KC eyes

*KC*= keratoconus; *UHR-OCT*= ultra-high-resolution optical coherence tomography; *ML*= machine learning

Utilizing Fisher’s scoring system allowed us to understand each individual feature’s contribution to the classifier by displays and comparisons through a visualization tool. Using the Scheimpflug-based camera alone, the maximum elevation value had the greatest influence on the neural network classifier. Although the diagnostic value of posterior surface variables from Scheimpflug-based cameras remains controversial [29], the importance of the variables extracted from the posterior elevation map of Scheimpflug-based cameras for screening is well recognized [18, 29–31]. Some studies showed that some variables, such as BAD-D from Pentacam, exhibited good performance in discriminating subclinical KC eyes from normal eyes [32]; a possible reason is that BAD-D utilized a regression model combined with some variables in elevations of both anterior and posterior corneal surfaces, corneal thickness, location of the thinnest point, Kmax, pachymetric regression and Ambrosio relational thickness [33]. But Hwang et al. implied BAD-D and similar individual metrics did not perform well enough to accurately distinguish subclinical KC eyes from a normal cohort [8]. This further implies that a model that combines more corneal parameters will assist clinicians in discriminating subclinical KC eyes from normal eyes. The elevation map of our study was based on an 8 mm best-fit sphere (BFS). Some investigators have noted that the diagnostic value of an elevation map based on an 8 mm enhanced BFS [31] or best-fit toric ellipsoid [34] should be considered, and future studies based on these elevation variables should also be considered. When using the UHR-OCT system alone, the EPV had the greatest influence on the neural network classifier, which echoed previous studies using epithelium thickness maps from OCT [6, 8]. The BPV also influenced the classifier, which echoed the results of several studies showing that irregularities in the Bowman layer can improve the detection of subclinical KC [35, 36]. The lack of automated Bowman’s layer analysis in a commercial anterior-segment OCT decreases awareness of the early change of Bowman’s layer in subclinical KC patients. This may result from the fact that current commercial anterior-segment OCT does not have enough bandwidth to detect the earliest changes with KC occurring at the level of Bowman’s layer, even with our UHR-OCT system. Future technical developments, such as new UHR-OCTs at an axial resolution of 1.5 μm level [37, 38] combined with 3D Bowman’s layer topography [39, 40] and deep learning automated corneal segmentation techniques [41], can help scientists and clinicians detect the true earliest change of the Bowman’s layer in subclinical KC patients.

Furthermore, the complexity of the subclinical KC screening system precludes reliance on a single machine, and the combination of clinical image modalities is the ultimate goal. Interestingly, when combining the Scheimpflug-based camera with UHR-OCT, the elevation variable from the Scheimpflug-based camera has superior differential ability compared with the UHR-OCT variables for screening KC eyes, but for subclinical KC eyes, the UHR-OCT-based single variable contributed more than the integrated variables from the Scheimpflug-based camera (ISV, IHD, etc.). The abundant depth information extracted from the epithelium detected by UHR-OCT contributed greatly to this finding, which indicated that segmented corneal layer information has great value in the diagnosis of subclinical KC but may be underutilized in clinical practice. There are some reasons for this phenomenon: first, the high cost of UHR-OCT restricts its application to clinics; second, clinicians cannot easily understand this information because of the lack of interpretation of segmented cornea layer information; third, automated segmented cornea layer (including epithelium and Bowman’s layer) software is not applicable to most commercial OCT systems.

Our study has several limitations. First, we used cross-validation in this study, and further study involving human experienced expert validation is needed. Second, the sample size of our study was limited, and further larger-scale studies are needed to validate our results. Third, we only used image modality features for the screening system, and whether biomechanical variables contributed to the system is still unknown. Fourth, we only tested parts of commonly used variables, and a study of more variables to assess overfitting is needed. Fifth, we only recruited subclinical KC and KC patients in this study, and our model was limited only to this disease. Future plans to recruit patients with additional corneal anomalies, such as post-Lasik ectasia and corneal warpage, could enhance our model. Sixth, our current model lacked comparison results with Pentacam indices such as PRFI and BAD-D, further studies using Pentacam with the latest software version can further explore machine learning models and comparisons with these indices.

## Conclusion

In conclusion, our study highlighted the value of combined instrument features from Scheimpflug-based cameras and UHR-OCT. These findings suggested that combined variables demonstrated better differential power than single-instrument variables. Furthermore, the UHR-OCT features showed superior value compared with the Scheimpflug-based camera features when differentiating subclinical KC eyes from normal eyes. The machine learning classifier could be a powerful automated screening tool for subclinical KC identification. We believe that our findings will direct future studies toward the best discrimination utilizing machine learning classifiers and multiple instrument-based features.

## Supplementary information

**Additional file 1:** **Figure S1.**The topographic maps of the subclinical KC eyes with max and min of I-S values. I-S: absolute value of the average curvature of inferior hemisphere minus superior hemisphere.

**Additional file 2: Figure S2.** The topographic maps of the subclinical KC eyes with max and min of IVA values. IVA: index of vertical asymmetry.

**Additional file 3: Table S1.** Demographics and top 10 variables of training and validation groups of three validation times using Pentacam HR model and UHR-OCT model to discriminate subclinical KC group from normal group.

## Data Availability

Not applicable.
