# Supplementary figures and images for: Machine learning helps improve diagnostic ability of subclinical keratoconus using Scheimpflug and OCT imaging modalities
Source: Eye Vis (Lond). 2020 Sep 10;7:48. doi: 10.1186/s40662-020-00213-3 (PMC7507244; doi:10.1186/s40662-020-00213-3)

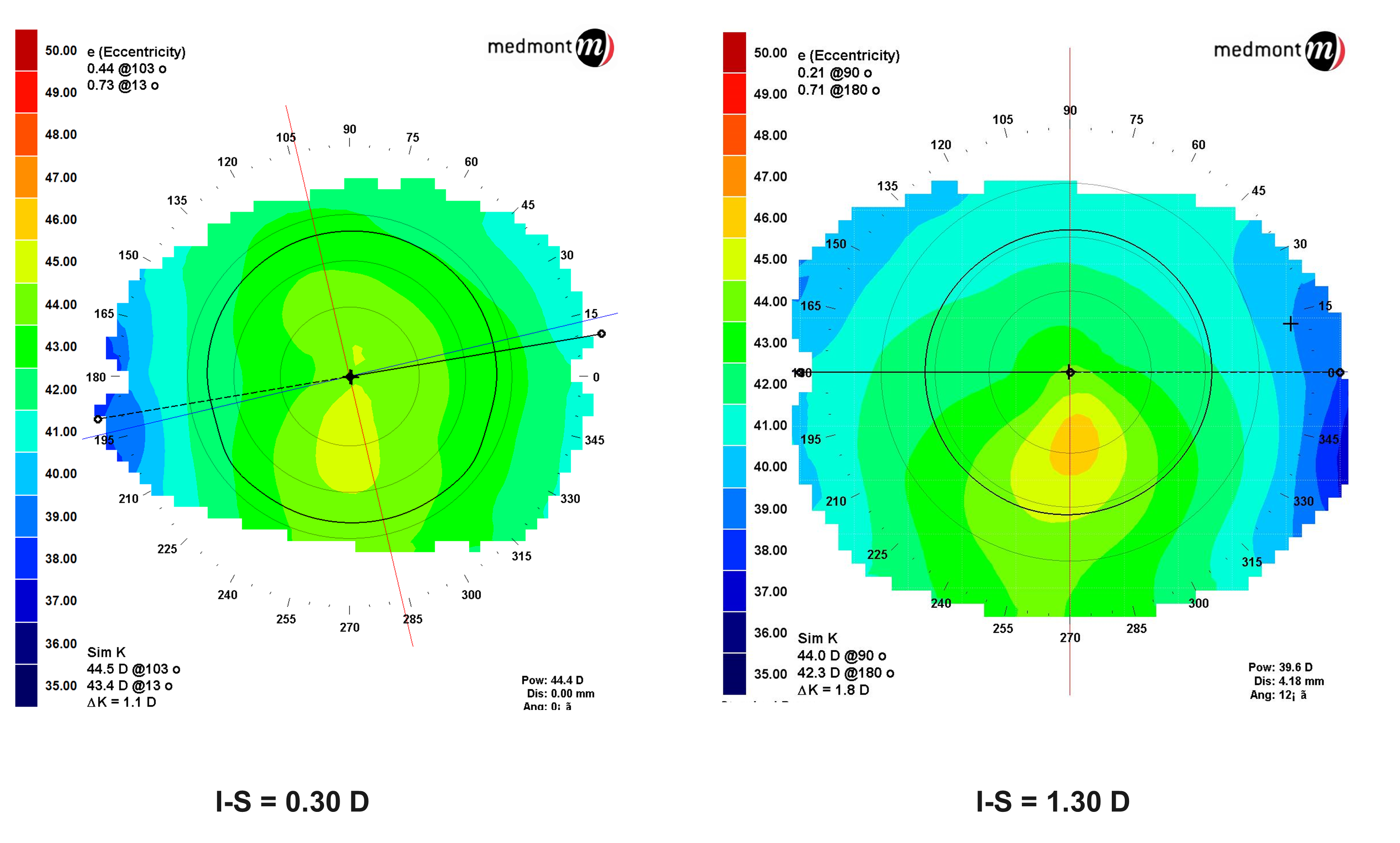

Supplement: Supplementary file 1 — Additional file 1: Figure S1.The topographic maps of the subclinical KC eyes with max and min of I-S values. I-S: absolute value of the average curvature of inferior hemisphere minus superior hemisphere. [file 40662_2020_213_MOESM1_ESM.tif]

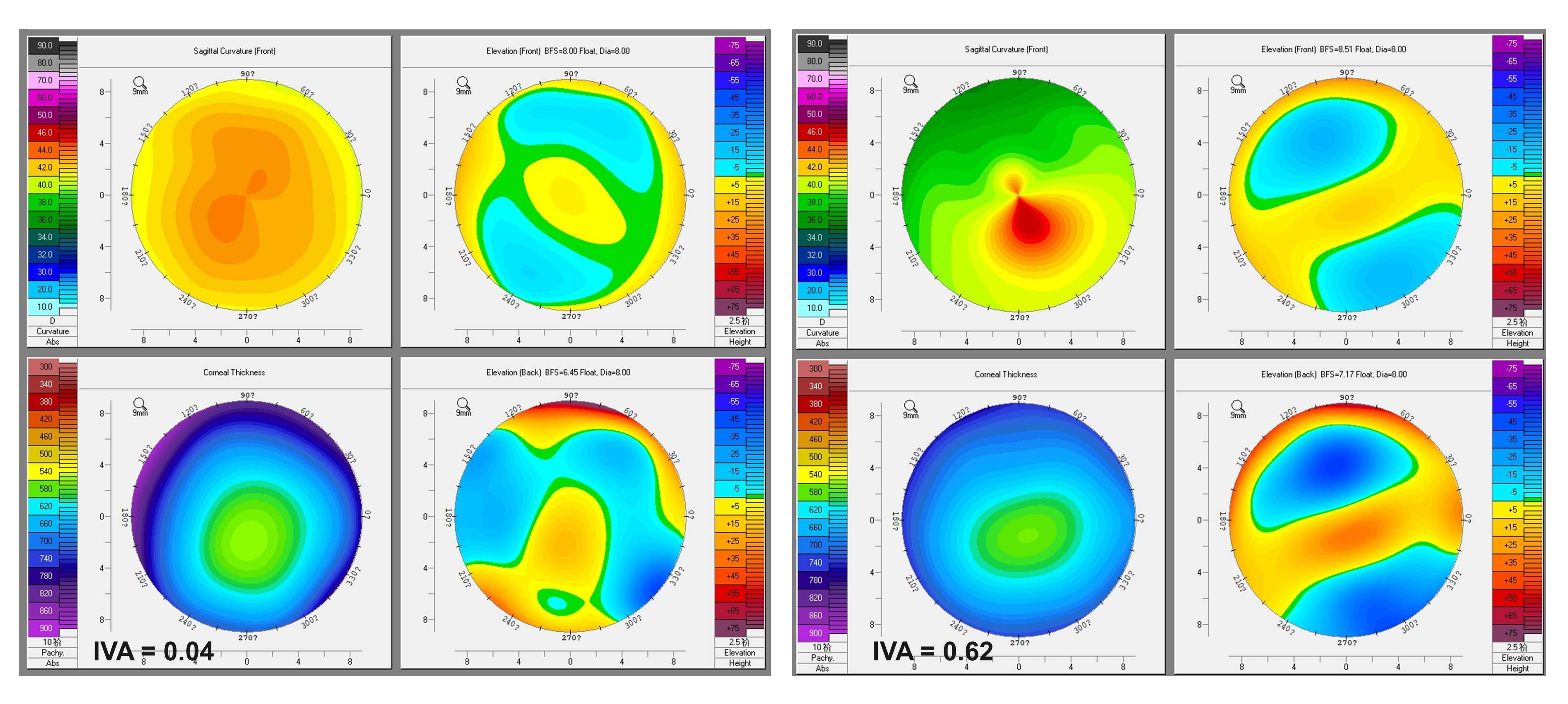

Supplement: Supplementary file 2 — Additional file 2: Figure S2. The topographic maps of the subclinical KC eyes with max and min of IVA values. IVA: index of vertical asymmetry. [file 40662_2020_213_MOESM2_ESM.tif]
