# Supplementary material for: Machine learning helps improve diagnostic ability of subclinical keratoconus using Scheimpflug and OCT imaging modalities
Source: Eye Vis (Lond). 2020 Sep 10;7:48. doi: 10.1186/s40662-020-00213-3 (PMC7507244; doi:10.1186/s40662-020-00213-3)
Supplement: Supplementary file 3 — Additional file 3: Table S1. Demographics and top 10 variables of training and validation groups of three validation times using Pentacam HR model and UHR-OCT model to discriminate subclinical KC group from normal group. [file 40662_2020_213_MOESM3_ESM.docx]

**Supplementary Table1: Demographics and top 10 variables of training and validation groups of three validation times using Pentacam HR model and UHR-OCT model to discriminate subclinical KC group from normal group.**

| Test Time | 1st | | | | | | | | 2nd | | | | | | | | | | | 3rd | | | | | | | | | | |
| --- | --- | --- | --- | --- | --- | --- | --- | --- | --- | --- | --- | --- | --- | --- | --- | --- | --- | --- | --- | --- | --- | --- | --- | --- | --- | --- | --- | --- | --- | --- |
| Group | Training | | | | Validation | | | | Training | | | | | Validation | | | | | | Training | | | | | | Validation | | | | |
|  | Normal | | Sub-KC | | Normal | | Sub-KC | | Normal | | Sub-KC | | | Normal | | | Sub-KC | | | Normal | | | Sub-KC | | | Normal | | | Sub-KC | |
|  | Mean | SD | Mean | SD | Mean | SD | Mean | SD | Mean | SD | Mean | SD | Mean | | SD | Mean | | SD | Mean | | SD | Mean | | SD | Mean | | SD | Mean | | SD |
| **Demographics** |  |  |  |  |  |  |  |  |  |  |  |  |  | |  |  | |  |  | |  |  | |  |  | |  |  | |  |
| SE(D) | -4.36 | 1.86 | -4.56 | 4.59 | -3.86 | 2.25 | -2.81 | 1.93 | -3.63 | 2.06 | -4.05 | 4.54 | -4.72 | | 3.24 | -3.98 | | 1.97 | -4.13 | | 2.2 | -4.5 | | 4.51 | -2.5 | | 1.76 | -3.63 | | 1.84 |
| BCVA (decimal VA) | 1.08 | 0.1 | 0.95 | 0.23 | 1.04 | 0.08 | 0.97 | 0.15 | 1.06 | 0.09 | 0.92 | 0.21 | 1.07 | | 0.09 | 1.1 | | 0.1 | 1.07 | | 0.09 | 0.94 | | 0.22 | 1.09 | | 1 | 0.92 | | 0.15 |
| Max-K (D) | 43.22 | 1.25 | 43.4 | 1.89 | 42.24 | 1.24 | 42.98 | 2.05 | 43.03 | 1.31 | 43.53 | 2.16 | 43.42 | | 0.91 | 42.55 | | 0.5 | 42.99 | | 1.18 | 43.5 | | 2.14 | 42.19 | | 1.51 | 42.6 | | 0.66 |
| Min-K (D) | 44.43 | 1.41 | 44.88 | 2.31 | 43.75 | 1.82 | 44.59 | 2.51 | 44.47 | 1.64 | 45.07 | 2.53 | 43.58 | | 0.97 | 43.98 | | 0.96 | 44.4 | | 43.7 | 45.1 | | 2.6 | 43.1 | | 1.68 | 44.43 | | 1.07 |
| Avg-K (D) | 43.83 | 1.31 | 44.24 | 2.09 | 43 | 1.45 | 43.78 | 2.27 | 43.75 | 1.43 | 44.39 | 2.3 | 43 | | 0.92 | 43.27 | | 0.69 | 43.7 | | 1.25 | 44.38 | | 2.35 | 42.64 | | 1.59 | 43.52 | | 0.4 |
| Ast-K (D) | 1.21 | 0.51 | 1.47 | 0.83 | 1.52 | 1.17 | 1.61 | 0.61 | 1.44 | 0.82 | 1.54 | 0.98 | 1.16 | | 0.42 | 1.43 | | 0.67 | 1.41 | | 78.9 | 1.61 | | 0.79 | 0.91 | | 0.38 | 1.83 | | 1.59 |
| **Top variables** |  |  |  |  |  |  |  |  |  |  |  |  |  | |  |  | |  |  | |  |  | |  |  | |  |  | |  |
| ISV | 16.93 | 5.79 | 23 | 7.54 | 17.73 | 7.48 | 24.56 | 7.04 | 17.97 | 6.41 | 24.04 | 7.58 | 15.56 | | 4.3 | 20.17 | | 4.49 | 16.87 | | 5.73 | 24.3 | | 7.24 | 15.86 | | 3.6 | 23.33 | | 7.97 |
| IVA | 0.13 | 0.07 | 0.2 | 0.1 | 0.14 | 0.06 | 0.23 | 0.11 | 0.13 | 0.07 | 0.22 | 0.11 | 0.1 | | 0.03 | 0.15 | | 0.03 | 0.12 | | 0.06 | 0.22 | | 0.1 | 1.36 | | 0.03 | 0.18 | | 0.12 |
| KI | 1.02 | 0.03 | 1.05 | 0.03 | 1.02 | 0.02 | 1.05 | 0.03 | 1.02 | 0.03 | 1.05 | 0.03 | 1.01 | | 0.02 | 1.04 | | 0.02 | 1.02 | | 0.03 | 1.05 | | 0.03 | 1.04 | | 0.02 | 1.05 | | 0.02 |
| Ecenter (back) (mm) | -0.78 | 2.28 | 2.33 | 6.11 | -1.09 | 1.54 | 0.49 | 1.83 | -0.44 | 2.08 | 1.41 | 4.4 | 0.07 | | 2.69 | 1.22 | | 3.08 | -0.08 | | 2.2 | 2.31 | | 5.68 | -0.29 | | 2.76 | 0.67 | | 1.54 |
| EMax(back) (mm) | 4.98 | 2.06 | 12.54 | 8.28 | 5 | 2.15 | 7.59 | 4.81 | 4.94 | 2.26 | 10.86 | 5.94 | 4.96 | | 1.98 | 6.94 | | 5.13 | 4.96 | | 3.46 | 11.69 | | 7.47 | 5.19 | | 3.52 | 10 | | 6.79 |
| EPSD （μm） | 3.22 | 1.2 | 4.29 | 1.21 | 3.02 | 0.7 | 4.87 | 1.43 | 3.2 | 0.96 | 4.39 | 1.23 | 3.48 | | 0.94 | 4.6 | | 1.45 | 3.46 | | 1.19 | 4.51 | | 1.22 | 2.9 | | 1.02 | 4.37 | | 1.44 |
| EPV (μm) | 2.94 | 0.62 | 4.12 | 1.13 | 2.73 | 0.73 | 4.06 | 0.77 | 2.8 | 0.75 | 4.09 | 1.04 | 3.03 | | 0.56 | 4.05 | | 0.85 | 2.75 | | 0.71 | 4.11 | | 0.9 | 2.88 | | 0.41 | 4.3 | | 1.1 |
| BPV (μm) | 1.24 | 0.26 | 1.73 | 0.37 | 1.36 | 0.23 | 1.82 | 0.45 | 1.21 | 0.23 | 1.66 | 0.39 | 1.37 | | 0.21 | 1.92 | | 0.53 | 1.26 | | 0.28 | 1.67 | | 0.37 | 1.1 | | 0.16 | 1.79 | | 0.56 |
| BEI-MAX （I/S）（μm） | 77.95 | 4.67 | 67.36 | 6.6 | 78.07 | 6.75 | 67.22 | 8.21 | 78.41 | 4.91 | 63.72 | 27.3 | 78.1 | | 5.91 | 65.2 | | 8.68 | 77.44 | | 6.03 | 63.02 | | 26.72 | 81.15 | | 4.57 | 67.26 | | 8.86 |
| BMIN (I) (μm) | 15.01 | 1.74 | 13.03 | 1.7 | 15.27 | 1 | 13.27 | 2.27 | 15.27 | 1.44 | 12.56 | 5.32 | 14.77 | | 1.33 | 12.15 | | 1.34 | 15.13 | | 1.56 | 12.22 | | 5.16 | 15.12 | | 1.21 | 13.39 | | 1.93 |
| Result | Logistic Regression: AUC: 0.91 Sensitivity: 100% 1-Specificity: 91%  Neural Network: AUC: 1.00 Sensitivity: 100% 1-Specificity: 100% | | | | | | | | Logistic Regression: AUC: 0.67 Sensitivity: 67% 1-Specificity: 100%  Neural Network: AUC: 0.83 Sensitivity: 100% 1-Specificity: 83% | | | | | | | | | | Logistic Regression: AUC: 1.00 Sensitivity: 100% 1-Specificity: 100%  Neural Network: AUC: 1.00 Sensitivity: 100% 1-Specificity: 83% | | | | | | | | | | | |

SE, spherical equivalent; BCVA, best corrected visual acuity; Max-K, maximum keratometry; Min-K, minimum keratometry; Avg-K, average keratometry; Ast-K, astigmatic keratometry; VA, visual acuity; D, diopter; EPV: Profile variation of epithelium; BPV: Profile variation of Bowman’s layer; Emax (back): Max elevation of 5 mm best-fit sphere of back corneal surface. ISV: Index of Surface Variance; KI: Keratoconus Index; IVA: Index of Vertical Asymmetry ; Ecenter (back): Central elevation of 5 mm best-fit sphere of back corneal surface; EPSD: epithelium profile standard deviation; BEI-MAX: Maximum ectasia index of Bowman’s layer; Bmin (I): the thinnest thickness of the inferior Bowman’s layer thickness map.
